# Anatomico-Chirurgical Observations on Dislocations of the Astragalus

**Published:** 1844-07

**Authors:** 


### Art. X.

1. *Anatomico-Chirurgical Observations on Dislocations of the Astragalus.* By Thomas Turner, Esq., m.r.c.s.l.—*Worcester,* 1843. 8vo, pp. 138.

2. *Mémoire sur l'Extirpation de l'Astragale, lu à l'Académie des Sciences dans la Séance du* 13 *Février,* 1843. Par MM. Rognetta et Fournier-Deschamps, d.m.

*A Memoir on Extirpation of the Astragalus; read before the Academy of Sciences,* 13*th Feb.* 1843. By MM. Rognetta and Fournier-Deschamps.

When reviewing the last volume of the 'Transactions of the Provincial Medical and Surgical Association,' we were compelled, from want of space, to pass by Mr. Turner's paper on Dislocations of the Astragalus, which is now published in a separate form. We take this, the earliest opportunity of supplying that omission; and shall, at the same time, advert to the memoir by MM. Rognetta and Fournier-Deschamps, mentioned at the head of this article.

Mr. Turner's object in the present treatise is "to assist in rescuing the surgeon from the painful dilemma in which he may be placed, for want of established rules to guide him in the arrangement and treatment of accidents occurring to the astragalus, respecting some of which we are left, by our best surgical authorities, in no inconsiderable degree of doubt and difficulty," (p. 7;) and he certainly adopted the best plan of attempting to remove whatever doubts and difficulties exist on the subject, by collecting and analysing recorded and unpublished cases, so as to deduce general inferences from a large number of facts. Accordingly, we are told that, "with a view to correct statistics, the author has made diligent search for published as well as unpublished cases," (p. 79,) and has accumulated forty-five; being "all the cases of dislocation of the astragalus which the author has, after much labour, been able to collect." (p. 78.) The work is therefore based on statistical research; and as completeness, copiousness, and accuracy of facts are the three things above all others needful in statistics, we must first inquire how far the author has complied with these essential requisites. We feel constrained to say, that his facts are neither complete nor, so far as they go, always accurate; for though we have to thank him for adding no fewer than sixteen unedited cases to those already on record, we can hardly admit that he has shown sufficient diligence in collecting, or correctness in quoting, the cases already known. It is, of course, but right that we should show cause for expressing this opinion; and, in attempting to do so we shall only notice omitted cases which are contained in books referred to by Mr. Turner himself, together with a few very generally known, either because of their importance, or of the celebrity of the works in which they are to be found. Mr. Turner refers to but twenty-nine published cases: we cannot pretend to say how many may exist in the books, but we conjecture they must amount to at least three times that number; but as we are not about compiling a statistical monograph on the accident in question, we must confine ourselves within the very limited field of inquiry just indicated.

In the last edition of Sir A. Cooper's work on 'Dislocations and Fractures of the Joints,' fourteen cases of dislocation of the astragalus are mentioned; four copied from other works, and ten either original or communicated to the author. Of these fourteen cases Mr. Turner quotes but eight, and consequently omits six; all of which were compound dislocations: three were reduced and recovered, (pp. 328-9-36;) one was kept unreduced, the bone sloughed out, and the patient recovered, (p. 328;) and, in the remaining two, excision of the astragalus succeeded in one case (p. 328), and terminated fatally in the other. (p. 331.) Before going further, we may observe: 1st. That the omission of Sir A. Cooper's 200th case (pp. 336-7) is the more to be regretted, as it is an example of an exceedingly rare form of dislocation of the astragalus not noticed at all by Mr. Turner; that, namely, in which the bone is displaced from the os calcis and os naviculare, while its connexion with the tibia and fibula remained undisturbed. We believe there are very few cases of the kind on record; and we are ourselves only acquainted with another, which is recorded by Dr. Macdonald. (Dublin Journ. of Med. Sc., vol. xiv, p. 235.) 2d. Though Mr. Turner, at p. 79, questions the truth of Sir A. Cooper's dictum, that "a simple dislocation of the astragalus sometimes, though rarely, occurs, a compound dislocation is still more rare;" yet he fails to observe that Sir A. Cooper's own work contains evidence, as far as it goes, in favour of Mr. Turner's doubts; for, of the fourteen cases therein recorded, but four are simple, and ten are compound. 3. Sir A. Cooper's 195th case (p. 130) is inaccurately quoted: it is Mr. Turner's seventh case, and, it is said, presents "a perfect illustration of compound dislocation forwards." (p. 40.) Whereas, the astragalus was dislocated *inwards;* and, further, the important fact is not mentioned that the posterior tibial artery was ruptured, and the accompanying nerve partially torn, and yet excision of the astragalus terminated very favorably. Mr. Hey, in his celebrated 'Practical Observations on Surgery,' (p. 388 et seq.,) mentions four cases of compound dislocation of the astragalus: in the first and fourth the astragalus was excised successfully; in the second, excision of that bone terminated fatally; and in the third, the patient having refused to suffer the projecting portion of bone to be cut away, the astragalus sloughed out. Not one of these cases is referred to by Mr. Turner; but only three of them can be reckoned here, as Hey's first case, which occurred in the practice of Mr. Trye, of Gloucester, is quoted by Sir A. Cooper, and is included amongst those just adverted to. The omission of Mr. Hey's fourth case (p. 386) is the more unfortunate, as it is expressly stated, that some motion remained in the ankle-joint, though the head of the astragalus was not extracted; and a simple reference to this and several other similar cases would have saved Mr. Turner the trouble of devoting eleven pages (pp. 126-36) to prove that Boyer and several others are wrong in supposing that excision of the astragalus must always terminate in anchylosis. Boyer cites twelve cases of dislocation of the astragalus, of which Mr. Turner quotes but seven. Amongst those he does refer to, some are very inaccurately given: thus, Mr. Turner's thirteenth case (p. 42) is Boyer's last case, (Traité de Malad. Chirurg. t. iv, p. 405;) and is given by our author as an example of dislocation of the astragalus "*forwards and inwards;*" whereas, it is expressly described by Boyer as an "*incomplete* luxation of the *head* of the astragalus *upwards* (not forwards) and *inwards.*" We are the more surprised at this mistake, as this case of Boyer's is remarkable for being the *only known* instance, as we believe, of this peculiar displacement; and thus we have a second rare form of dislocation of the astragalus totally unnoticed by Mr. Turner. But this case involves a series of mistakes: its issue is stated in Table II (p. 85) to have been "deformity and permanent lameness;" but at p. 42 it is more correctly stated, that ultimately there was no lameness, though the deformity (it should be *slight* deformity) continued. Again, Mr. Turner, from confounding this *comparatively* unimportant accident with the much more serious one of dislocation of the astragalus *forwards,* is led (in the section on the consequences of leaving dislocations of the astragalus unreduced,) to say, that as the motions of the foot ultimately become free, this case confirms the belief that in simple and partial irreducible dislocations of the astragalus, either forwards, outwards, or inwards, we should not interfere, but leave the case to the resources of nature. We quite agree in the rule of practice, as a general rule; but if it had no better foundation than the case appealed to in its support, we fear it could hardly be maintained. As another, but, we admit, a trifling example of inaccuracy, we may observe that Daniel's case is given as if it occurred in Boyer's practice. Such trifles, however, give no small trouble to the scrupulous inquirer; especially as Boyer's work, from which the latter case seems quoted, is not referred to; and we may further remark, that Mr. Turner is so very loose in and sparing of his references, that it is often extremely difficult to identify the cases he quotes, and to discriminate those that he omits to quote. So much confusion, indeed, arises from the way in which the cases are given, that it was only while penning these lines we discovered another mistake with reference to Boyer. Mr. Turner's forty-second case is given as an example of dislocation of the astragalus backwards; and purports to be extracted from Boyer's works. We were positively certain that Boyer had recorded no such accident; but after some research we discovered the case alluded to by Mr. Turner, (Boyer, p. 403;) which is not an example of dislocation of the *astragalus* backwards, but of the *entire foot* backwards; the connexions and relations of the astragalus to the tarsus remaining unaltered—an accident which Sir A. Cooper names dislocation of the ankle-joint, or of the tibia, forward, but which most writers term dislocation of the foot, and which no author since the days of B. Bell considers as a dislocation of the astragalus, and which is utterly unlike the other cases adduced by Mr. Turner himself, which are really displacements of the astragalus from the os calcis and scaphoid bone. It is not a little curious to observe with what very different views Boyer and Mr. Turner adduce this case. Boyer says that the signs of dislocation of the foot backwards "are *so evident* that a mistake respecting the accident *would be very difficult;* nevertheless, there is an example of such a mistake having been committed," (p. 403;) and then he gives the case, "the history of which" —we now quote from Mr. Turner—"illustrates the fact, that dislocation backwards, owing to the space being so considerable between the posterior part of the tibia and tendo Achillis, *may be overlooked;* a mistake scarcely likely to happen in dislocation in any other direction." (pp. 74-5.) This observation, of course, arises from the error as to the real nature of the case commented on: and here we must observe, that, deducting this case, as not being on dislocation of the astragalus, Mr. Turner quotes six cases from Boyer, and not seven, as we stated above, and the sum total of his cases is reduced to forty-four. Among the cases quoted by Boyer, but overlooked by Mr. Turner in his statistics, though incidentally mentioned elsewhere, is one of successful extirpation of the astragalus by F. Hildanus, celebrated as being the first example of the operation on record. The remaining five belong to Dessault, who has further recorded two others, making seven in all, which may be found in his 'Œuvres Chirurgicales,' by Bichat, (t. i, p. 435, et seq.) and 'Journ. de Chirurg.,' (t. i;) but not one of those well-known cases is quoted by Mr. Turner. Of Dessault's cases, two were simple, and one was reduced with facility; but it is a remarkable fact in the history of this accident, that the second being irreducible, Dessault attributed the failure to the smallness of the opening made in the capsular ligament by the head of the astragalus, and freely divided the integuments and the ligaments; after which the dislocation was easily reduced, and the case did well. Nor is this an isolated case; for the same practice, we may here observe, was successfully adopted, under similar circumstances, by Navula. (Bull. de la Fac. de Méd., 1812.) The five other cases mentioned by Dessault were all compound, and in all the astragalus was excised: four of the patients recovered and but one died; not, indeed, it would appear, in consequence of the accident, but yet it is safer to reckon this among the fatal cases. We can the less account for Mr. Turner's neglect of Dessault's cases, as he actually incidentally alludes to two of them, (pp. 93, 131,) which, we suppose, caught his eye in turning over Boyer, for they are both mentioned by that author: but, we repeat, they are only incidentally alluded to, and are not included in the cases for which our author made "diligent search" "with a view to correct statistics." Dupuytren is said to have met with ten or twelve cases of dislocation of the astragalus, but only five of them are distinctly recorded in his works; one in his 'Mémoire sur les Fractures du Peroné;' and four in the 'Leçons Orales.' Of these five cases, Mr. Turner quotes but three, and contrives to involve them in almost inextricable obscurity. The first case cited from Dupuytren is Mr. Turner's fifth, (p. 38,) and is most remarkable as one of the very few instances on record in which a *simply* luxated astragalus was cut down on *immediately* and extirpated. The reference given for this case at p. 38 is the first volume of the 'Medico-Chirurgical Journal;' but it *must* be the case recorded in the above-mentioned memoir by Dupuytren, published in the 'Ann. des Hôp. de Paris,' as no other similar case has been published as occurring in Dupuytren's practice; yet, strange to say, we find this very case again incidentally mentioned by Mr. Turner (p. 53), where the correct reference to the original memoir is given. But this is not all. At p. 39 we are rightly told that "the bone was cut away," but it is added that the result was recovery, "with anchylosis of the joint;" and yet, in Table II, facing p. 85, which purports to give a "*classification of treatment, and results of dislocations of the astragalus*," this very case (No. 5), in which the bone was cut away, is enumerated amongst those headed, "*Bone allowed to remain in its new situation*;" and the result is again stated to be anchylosis of the joint. We need not stop to point out the contradictory statements respecting the treatment of the case contained in the text and in the table; but we must observe that there is not one word in the original memoir warranting the averment that anchylosis occurred: we are merely told that, three years after the accident, the patient walked "avec *un peu* de claudication, il est vrai, mais d'ailleurs aussi vite et aussi longtemps qu'avant son accident." The twenty-second case in the present work is also inaccurately given. "Reduction," it is said, "was attempted, but without success." (p. 49.) But the fact is, Dupuytren nearly, though not completely, replaced the bone. This twenty-second case is therefore wrongly classified in the table already referred to, as it should have been placed under the head, "partial reduction." Furthermore, as already stated, two of Dupuytren's cases are totally omitted; one simple reduced, the other compound and irreducible, the wound having healed, the patient applied to Dupuytren six months after the accident, because of the deformity and uselessness of the foot, the astragalus then was excised, and the patient obtained a very useful foot.

We have now referred to twenty-two cases, which are all, save two, contained in the works of Cooper, Hey, Dessault, Boyer, and Dupuytren, and have also, we think, abundantly shown how imperfectly the facts borrowed from some of those authors have been dealt with. Many more cases exist in the books; we do not pretend to be in a predicament to cite them all, or even a small portion of them; nor, if we were, would this be the place to do so; but we shall print a few that we do happen to know, all of which bear on more or less important points of the history of the accident. De Guignieres (Bull. de la Fac. Méd. de Paris, t. iii, p. 238,) records a case of compound dislocation of the astragalus inwards, with fracture of the fibula. The astragalus was excised, and the patient recovered *without anchylosis*, a *new joint* having formed between the tibia and the os calcis; a circumstance which we thus particularize for a reason already mentioned. To this case there is appended one by Chaussier, of irreducible compound dislocation of the astragalus forwards, in which, as the resistance of the tendons was the chief obstacle to reduction, the tendons of the tibialis anticus, the tibialis posticus, and the extensor proprius pollicis pedis were divided; the dislocation was then easily reduced; but the patient died. No doubt the practice adopted here is by no means analogous to the modern application of tenotomy; but still the case deserves to be mentioned in connexion with that subject, for it at least shows that division of the tendons facilitates reduction. Follot (Archiv. Gén. de Méd., t. xx, p. 292) relates a case—or rather, there is a report on the case—of complete compound luxation and comminuted fracture of the astragalus, with rupture of the tendon of the tibialis anticus muscle; but, notwithstanding the immense violence sustained, and the great injury to the soft parts, extirpation of the astragalus terminated in cure by anchylosis, rendering the case one of the most important on record in favour of the practice adopted, as compared with amputation. M. Barbieux also successfully removed the astragalus on the second day, in compound dislocation of that bone. (Journ. Complm. du Dict. des Scienc. Méd., t. ix, p. 285.) Again, M. Rey gives a case of complete compound dislocation inwards, and semi-rotation of the astragalus, and partial dislocation of the tibia on the astragalus forwards, *without fracture* of either bone: excision of the astragalus terminated in recovery. (Gaz. des Hôp., t. x, p. 470.) Mr. Norris of Pennsylvania, in a case of *simple* irreducible luxation of the astragalus forwards and outwards, as the integuments over the bone were very tense and discoloured, cut down on and removed it, excepting its head, which, being broken, was left adhering to the os naviculare. Gangrene supervened, and amputation terminated in death. This is the only case we know of in which the practice adopted by Dupuytren, in one instance, of *immediately* excising a *simple* luxated astragalus, has been imitated; and we think the condition of the soft parts in each fully justified the operation. (Gaz. Méd. de Paris, 1837, p. 796.) Finally, Dupuytren being unable to reduce a simple dislocation of the astragalus, left it in situ; the limb gangrened and was amputated, but the patient recovered. On dissection, the astragalus was found rotated on the os calcis. (Archiv. Gén. de Méd., 1833, p. 506.)

We had intended to examine how far the cases we had added to those given by Mr. Turner, alter the various numerical results he has arrived at, but to do so fully would carry us too far, and, moreover, could not lead to anything conclusive, inasmuch as a statistical investigation should be founded on all, or some reasonable approach to all, the facts connected with the subject under inquiry. We shall therefore allude to one point only: Mr. Turner gives two to cases of *simple*, and twenty-one of *compound* dislocations of the astragalus, and is thence led to doubt the accuracy of Sir A. Cooper's opinion, that *compound* dislocation occurs more rarely than *simple* dislocation of that bone: the addition of the *unselected* cases we have referred to would lead to a result directly contrary to Sir A. Cooper's opinion, for the numbers then would be thirty-one *simple*, and forty-two *compound*. It remains to be seen, however, whether Sir A. Cooper's opinion may not prove true, if ever a very extensive series of cases is collected.

The mechanism of dislocations of the astragalus is as yet but imperfectly understood, and as the subject is important and interesting, we shall here notice M. Rognetta's experiments on the subject which originally appeared in the Archiv. gén. de Méd., 1833, t. iii, p. 485, and are in fact republished in the memoir mentioned at the commencement of this article.

When we inspect the astragalus in the skeleton, it appears most liable to be displaced inwards, because of the deep notch between the os calcis and the os naviculare, but in the living subject, primary displacement inwards must be difficult, or rather impossible: 1, Because the notch in question is filled by very powerful ligaments; 2, because the head of the astragalus can most readily escape directly upwards, as the upper part of the capsular ligament is loose and thin; and 3, because, according to M. Rognetta, the only power capable of dislodging the astragalus is the tibia acting as a lever of the first order, in the direction of the long axis of the foot. In walking, the astragalus may be considered a moveable pivot, on which a threefold motion takes place: 1, That of the bones of the leg on the pulley of the astragalus; 2, that of the astragalus itself on the bones of the tarsus; and 3, that of the astragalus conjointly with the bones of the leg on the bones of the tarsus. Now there is no lateral motion between the astragalus and the tibia, and fibula, and inversion and eversion of the foot are exclusively effected in the joints of the tarsus, but the 3d, or conjoint motion above referred to, is more complex.

When the leg is raised from the ground to take a step, it is first slightly flexed on the foot, and the foot is simultaneously flexed on the leg, so that there is a double inverse motion, that of the pulley of the astragalus backwards, and that of the articular surface of the tibia forwards. But when the whole foot is flexed, and the pulley of the astragalus rotated backwards, the head of that bone must be depressed in the scaphoid cavity, towards the sole of the foot. When the foot is now thrown forwards, it is extended on the leg, and the leg is extended both on the foot and on the thigh, and now it is that the conjoint motion of the bones of the leg with the astragalus comes into play. The tibia bearing on the astragalus posteriorly depresses it in that situation, and the head of the astragalus consequently becomes more or less prominent on the dorsum of the foot. That this is the case is easily ascertained by placing the fingers over the head of the astragalus while an individual is walking, when it will be felt elevated or depressed, according as the foot is extended or flexed on the leg. Hence it follows that when the foot and leg are both forcibly and simultaneously extended, the head of the astragalus projects towards the dorsum of the foot, has a tendency in fact to be dislocated from the os naviculare; and, generally speaking, the conditions necessary for the occurrence of dislocation of the astragalus, are that the foot shall be forcibly extended on the leg, and the leg powerfully extended on the foot by the weight of the body; the luxating force impinges on the astragalus entirely through the tibia, which acts as a lever of the first order, the power being applied at its upper extremity, the resistance being at the astragalus and the os calcis, constituting the fulcrum, but the astragalus can scarcely be dislocated unless the tibia acts in the direction of the antero-posterior axis of the foot, for if either malleolus acts singly or obliquely, and tends to displace the astragalus laterally, the malleolus itself will first give way.

M. Rognetta, with the object of illustrating those views, performed a series of experiments, which, though analogous to those instituted by Dupuytren, require to be noticed, both because they more especially relate to the tarsal articulation of the astragalus, and because his results are not quite in accordance with those obtained by Dupuytren.

When the foot of the dead subject is engaged between the bars of an iron grating, and the body is forcibly drawn back; M. Rognetta found that the tibia and fibula were easily fractured, and then the head of the astragalus ceased to project on the dorsum of the foot; in some young subjects, however, the tibia offered great resistance, but the fibula broke near the ankle, and then dislocation of the *pulley* of the astragalus and of the *foot* outwards could be effected, but dislocation of the *foot* inwards, or dislocation of the *astragalus* properly so called, could never be accomplished. M. Rognetta now repeated the experiments. Having denuded the bones of all the soft parts, except the ligaments, and, acting on the lever formed by the leg, gradually instead of forcibly and suddenly, observed that the anterior ligament of the ankle-joint first gave way; on continuing the extension, the external malleolus, which is larger than the internal, came in contact with the posterior part of the os calcis; further extension fractured the fibula, inferiorly and partially, or completely ruptured the ligaments that unite it to the tibia,—a circumstance which greatly facilitated the production of dislocation of the astragalus, because the tibia, when thus separated from the fibula, has only the resistance of the astragalus to overcome, and, except when the tibia gave way, the head of the astragalus, on continuing the extension, was always luxated forwards; once this effected, the astragalus could be easily thrown either outwards or inwards, but it was impossible to displace that bone laterally, before its head was thus thrown forwards, as the tibia or fibula respectively were always fractured in the attempt.

From these experiments M. Rognetta, among other conclusions, maintains that the astragalus must always be dislocated forwards before it can be displaced laterally. The extent of the luxation may of course vary infinitely from simple dislocation of the head of the astragalus upwards, (as occurred in Boyer's remarkable case already mentioned,) to complete dislocation forwards, or from the slightest to the most complete lateral displacement.

One of the most remarkable circumstances connected with dislocation of the astragalus is the occasional rotation of that bone on its axis, of which M. Rognetta offers the following ingenious explanation. If during a fall the foot is extended, the tibia pushes the astragalus forwards, and the head of the latter bone being thrown upwards from the scaphoid cavity, distends the integuments and tendons on the dorsum of the foot; the body of the astragalus being still impelled forwards, its head is now placed between the two opposite forces, that exerted by the tibia through the body of the bone, and that offered to it by the integuments, which, as they present more resistance towards the toes than on the dorsum of the foot, tend to throw the head of the astragalus backwards, and may thus cause complete rotation of the bone. We may thus understand how the astragalus has been rotated on its transverse axis, but M. Rognetta offers no explanation of the more difficult case, where the bone is rotated on its antero-posterior axis, and its head consequently remains in front.

This article has already exceeded the length we originally anticipated; but we cannot conclude without briefly considering the "principles of treatment in dislocations of the astragalus" laid down by Mr. Turner, (p. 88 et seq.) the more especially as we think they are, on the whole, very judicious, though in a few particulars we, perhaps erroneously, differ from the author.

*Reduction* should always be accomplished in partial and simple dislocation of the astragalus, but if it is impeded by the astragalus being twisted, &c.; operative interferencc should be abstained from, as the patient has a reasonable chance of obtaining one useful foot by trusting to the resources of nature. In *complete* and *simple* dislocation, the chief and too often insuperable obstacle to reduction is the approximation of the tibia to the os calcis by muscular action, which being effected by all the muscles passing from the leg to the foot, can be but partially overcome by flexing the leg on the thigh. Reduction, however, can sometimes be accomplished, especially when there is fracture of the leg, or of the os calcis, or dissevering of the bones of the tarsus, as the purchase of the muscles is then impaired, (p. 99;) but is it always safe to return the bone? This question Mr. Turner does not answer very explicitly, but seems to imply that when the bone is so loose as to be *easily* returned, it will be likely to die, and thus place the patient in a worse predicament than if it had been excised or left unreduced. (p. 92.) We doubt the wisdom of this precept, and at all events *if* we were so persuaded that the bone must die as to abstain from reducing it when practicable, we would greatly prefer excising it immediately. In complete compound dislocations of the astragalus alone, without fracture or solution of continuity and connexion in the bones and joints of the tarsus, all attempts at reduction are, in Mr. Turner's opinion, not only hopeless but prejudicial, (p. 96;) this rule may possibly be too absolute, but is, we believe, generally true. Mr. Turner admits tenotomy to be justifiable as a means of facilitating the reduction of partial dislocation, but speaks, we think, too doubtingly as to its applicability in complete luxation. (pp. 94-5.)

*Leaving the astragalus in its new situation.* In dislocation backwards, the bone scarcely seems to act as a foreign body, and the cases in which it was left unreduced did well. (pp. 92, 101.) In *partial* and *simple* dislocations, either forwards or laterally, we should not interfere either. If the bone is left unreduced in *complete but simple* dislocations, the bone is very apt to excite inflammation, &c., and to slough out, but still Mr. Turner would, if the bone was protruded *directly* in reference to it, leave the case to nature, and subsequently, if inflammation set in, act as specified in the section on excision.

*Excision of the astragalus.* In *partial* and compound dislocations, (reduction failing,) the *protruded* portion of the bone should, if practicable, be excised. In reference to complete excision of the astragalus, we shall borrow the author's words:

"It may be summarily stated, that in simple, direct, and complete luxation, the author advocates the practice of allowing the bone to remain in its new situation, without any operation, until it manifests a tendency to ulcerate the skin, in which case he would make an excision over the bone, to relieve tension and pressure, and that when the bone is so far detached from the circumjacent textures by the natural process of separation, he would remove it. In simple, indirect, and complete luxation, he would anticipate, as a matter of certainty, that the bone would die and require dislodgment; to take off tension and pressure from the angles of the displaced bone, he would at once make an excision over it, but not remove the bone, wishing to benefit by the probability that the exposure of the cavity of the joint may have an injurious effect. [*Sic* in orig.] In complete compound luxation, whether direct or indirect, or complicated with fracture or with dislocation of the ankle-joint, he would immediately proceed to the removal of the astragalus, from believing that the limb will be put in a better condition for the reparative process of the joint, by the abstraction of the processes of inflammation, suppuration, ulceration, and sloughing, (processes necessary to the disengagement of the astragalus by natural efforts.") (pp. 110-11.)

On this passage we have to observe that we differ from Mr. Turner in this respect only, that whenever we saw reason to cut down on a simply luxated astragalus, we would certainly proceed to remove the bone at once, if the case were one of partial dislocation; circumstances might indeed render it difficult to do so, but at all events we think the accessible portion of the bone should be removed. In a word, if we converted a simple into a compound dislocation, we would, as a general rule, deal with it precisely as if it had been compound in the first instance.

In conclusion, though we cannot compliment Mr. Turner on his diligence or accuracy in research, we have to thank him for an useful essay, which he could, we think, convert into an extremely valuable monograph by the adoption of more extended and accurate statistics, and a somewhat more methodical arrangement of his practical commentary.

